# Expression of Ik6 and Ik8 Isoforms and Their Association with Relapse and Death in Mexican Children with Acute Lymphoblastic Leukemia

**DOI:** 10.1371/journal.pone.0130756

**Published:** 2015-07-01

**Authors:** Adriana Reyes-León, Rocío Juárez-Velázquez, Alma Medrano-Hernández, Teresa Cuenca-Roldán, Consuelo Salas-Labadía, María del Pilar Navarrete-Meneses, Roberto Rivera-Luna, Gerardo López-Hernández, Rogelio Paredes-Aguilera, Patricia Pérez-Vera

**Affiliations:** 1 Laboratorio de Cultivo de Tejidos, Departamento de Genética Humana, Instituto Nacional de Pediatría, México, DF, México; 2 Subdirección de Hemato-Oncología, Instituto Nacional de Pediatría, México, DF, México; 3 Hospital para el Niño Poblano, Puebla, México; 4 Servicio de Hematología, Instituto Nacional de Pediatría, México, DF, México; University of Heidelberg, GERMANY

## Abstract

Expression of the 6 and 8 dominant-negative Ikaros isoforms in pediatric patients with acute lymphoblastic leukemia has been associated with a high risk of relapse and death; due to these isoforms disrupting the differentiation and proliferation of lymphoid cells. The aim of this study was to know the frequency of Ik6 and Ik8 in 113 Mexican ALL-children treated within the National Popular Medical Insurance Program to determine whether there was an association with relapse-free survival, event-free survival and overall survival, and to assess its usefulness in the initial stratification of patients. The expression of these isoforms was analyzed using specific primer sets and nested RT-PCR. The detected transcripts were classified according to the isoforms’s sizes reported. A non-expected band of 300 bp from one patient was analyzed by sequencing. Twenty-six patients expressed Ik6 and/or Ik8 and one of them expressed a variant of Ik8 denominated Ik8-deleted. Although the presence of them was not statistically associated with lower relapse free survival (*p* = 0.432), event free survival (*p* = 0.667) or overall survival (*p* = 0.531), inferior overall survival was observed in patients that expressed these isoforms and showed high or standard risk by age and white blood-cell count at diagnosis. Of the 26 patients Ik6+ and/or Ik8+, 14 did not present adverse events; from them 6 were exclusively Ik6+ and/or Ik8+, and 8 were positive for the other Ikaros isoforms (Ik1, Ik2, Ik5, Ik3A, Ik4, Ik4A, Ik7). In the patients studied, the expression of Ik6 and Ik8 did not constitute an independent prognostic factor for relapse or death related to disease; therefore, they could not be used in the initial risk stratification.

## Introduction

Treatment improvement in childhood acute lymphoblastic leukemia (ALL) depends on the assessment of conventional risk factors such as age <1 year or ≥10, white blood-cell (WBC) count ≥50x10^9^/L, detection of extramedullary disease, immunophenotype of T-cells, and presence of the *BCR-ABL1* fusion or *MLL* gene rearrangements, as well as on the detection of novel molecular markers of poor prognosis [1]. Gene expression analyses of lymphoblasts at diagnosis have identified several genes with significant prognostic potential [1]. One of them is the *IKZF1* gene located on chromosome band 7p12; it encodes Ikaros, a transcription factor that plays a critical role in the development of all lymphoid lineages [2,3]. Ikaros comprises 6 zinc finger domains in 7 exons: 4 of them into the exons 3, 4 and 5 at the N-terminal region, with DNA-binding activity; and 2 domains into the exon 7 at the C-terminal region, which are required for hetero and homodimerization among the Ikaros isoforms and interaction with other Ikaros proteins family [4]. Through alternative splicing and focal or broad deletions, *IKZF1* encodes 11 different isoforms [5–7]. Each isoform contains a specific set of zinc finger domains dictates differential DNA binding capacity. Five of these isoforms (Ik1, Ik2, Ik2A, Ik3 and Ik3A) are “long” and functional, because they conserve at least 3 DNA binding domains. The remaining isoforms are referred to as “short” (Ik4, Ik4A, Ik5, Ik6, Ik7 and Ik8) and have 2 or less DNA binding domains [3,6]. Particularly, Ik6 and Ik8 lack exons 3–5, are unable to bind DNA with high affinity, and do not activate transcription; therefore they act as dominant-negative isoforms (DNI) disrupting the differentiation and proliferation of lymphoid cells, besides participating in the leukemogenic potential [8,9]. The presence of DNI in leukemic blast cells confers hyperproliferation and resistance to apoptosis contributing to the development of leukemia [5,9]. In patients with ALL, these isoforms are associated with a significant increase in the frequency of relapse, resistance to chemotherapy and decrease in survival rates [10]. This observation is consistent with the fact that non-functional isoforms have been observed more frequently (63–80% of cases) in the subgroup of high-risk patients with *BCR-ABL1*+ [11]. Moreover, microarray gene expression studies have revealed that a group of *BCR-ABL1*- patients with *IKZF1* deletions shows similar profiles to those found in *BCR-ABL1*+ patients. The patients with *IKZF1*, *CRLF2* alterations and *JAK* mutations have recently been considered as a "BCR-ABL1-like" group, because they harbor novel kinase-activating mutations and present adverse prognostic features [7,8]. The Ikaros DNI have been considered as independent predictors of adverse outcome in pediatric ALL, but their prognostic impact seems to be different among cohorts [12–14]. In developed countries the advances in management of pediatric ALL have resulted in an increased cure rate of 76–86% [1], however, in countries like Mexico only some Institutions with specialized pediatric oncology units have current overall survival for ALL children of 75% [15]. Based on these results, it is important for us to identify genetic markers that could help to perform more suitable risk stratification. The early identification of Ikaros non-functional isoforms as a prognostic marker could benefit those patients who initially are not classified in the high-risk group based on other conventional risk factors. The aim of this study was to detect the DNI Ik6 and Ik8, in Mexican ALL-children treated with the Public Medical Insurance Program (PMIP) [16,17], to know their frequency and association with conventional prognostic factors and adverse events, as well as to evaluate its usefulness in the initial stratification of patients and future therapeutic application.

## Materials and Methods

### Selection of patients and samples

Pediatric patients with B-cell (B-ALL) and T-cell (T-ALL) ALL diagnosed at the Instituto Nacional de Pediatría and the Hospital para el Niño Poblano in Mexico, were consecutively recruited from February 2008 to August 2011. The project was approved and followed the guidelines of the Research and Institutional Ethics Committees (Instituto Nacional de Pediatría, approved project number 06/056). Patient’s parents or guardians signed informed written consent before bone marrow samples were collected at diagnosis. The clinical progression of the patients was followed-up. Samples from 113 patients were available for molecular analysis. Cases in which RNA could not be obtained, and patients who received treatment prior to the bone marrow collection were eliminated.

Leukemia was classified by immunophenotyping according to the following criteria: for B-ALL, cCD79a+, cCD22+ and CD19+ with additional analyses of CD34, TdT, CD10, CD20 and CD22; for T-ALL, cCD3+ and CD7+ with additional analysis of CD34, TdT, CD1, CD2, CD3 and CD5. The treatment protocols were based on the scheme of the PMIP for Mexican ALL-children that includes the following drugs: prednisone, L-asparaginase, vincristine, methotrexate, etoposide, cytosine arabinoside, 6-mercaptopurine, and doxorubicin [16,17].

### Expression of Ikaros isoforms

Mononuclear cells were obtained from bone marrow samples using density gradient centrifugation (Lymphoprep, Axis-Shield PoC AS, Oslo, Norway). The purified cells were suspended in lysis buffer and stored in liquid nitrogen until the time of extraction. Total RNA was extracted using the RNeasy mini kit (QIAGEN, Hilden, Germany) according to the manufacturer's instructions. cDNA was obtained by standard methods (Invitrogen, California, USA). Ikaros isoforms were amplified by nested RT-PCR using cDNA as template, as previously described [6]. The primer set for the first reaction was F1 5’-ATGGATGCTGACGAGGGTCAAGAC-3’ and R1 5’-TTAGCTCATGTGGAAGCGGTGCTC-3’; and the second reaction was performed with the primer set F2 5’-CCCCTGTAAGCGATACTCCAGATG-3’ and R2 5’-GATGGCTTGGTCCATCACGTGGGA-3’, the obtained products included all *IKZF1* transcripts. Amplification of *ABL1* gene was used as internal control and the primer set used was L-abl_F 5’-AAGGGGCTGTCCTCGTCCTC-3’ and L-abl_R 5’- CCAGGAGGTTCCCGTAGGTCA-3’. The PCR products were examined qualitatively by agarose gel electrophoresis (2.5% agarose gels), and bands were identified according to reported size in base pairs (bp) for each Ikaros isoform using a 100 pb DNA Ladder (Invitrogen, California, USA). The size of each isoforms were: Ik1 (893 bp), Ik3A (758 bp) and Ik2 (632 bp), that belong to the long functional isoform group, and Ik4 (505 bp), Ik5 (464 bp), Ik7 (464 bp), Ik4A (370 bp), Ik8 (337 bp) and Ik6 (202 bp) which conform the short non-functional isoforms [6]. All samples were analyzed by duplicate. The expression level for all isoform was semiquantitatively analyzed using the ImageStudioDigits software. Three groups of expression (high, medium, low and null) were designated through a scatter plot data. Twenty-two different expression patterns were observed. Sequencing analysis was performed in order to identify bands with unexpected sizes. The PCR products were cleaned using the QIAquick Gel Extraction Kit (QIAGEN, Hilden, Germany) and nucleotide sequencing was performed using the dideoxynucleotide chain termination method with a BigDye Terminator v3.1 cycle sequencing kit in an ABI PRISM 377 automated DNA sequencer (Applied Biosystems, California, USA). The nucleotide sequences and derived amino acid sequences were analyzed by Chromas software (version 1.62; Technelysium) and aligned with DNAMAN program (Version 3.0, Lynnon BioSoft). The obtained sequence from one patient was compared with the reference sequences of Ik8 and Ik8-deleted (GenBank accession numbers NM_001220775 and NM_001220776 respectively).

### Statistical analysis

Univariate analysis was carried out using central tendency tests to characterize the study sample. Fisher's exact test was applied to determine differences between the groups of patients who did and did not show relapse or death in terms of Ikaros isoforms and clinical-biological characteristics. A survival analysis was performed; relapse-free survival (RFS) was defined as the time elapsed from the date of diagnosis to the appearance of relapse. Event-free survival (EFS) was considered as the time passed from the diagnosis to the date of death in induction, therapy failure at remission induction, relapse or death in remission, or at diagnosis of a second malignant neoplasm. Overall survival (OS) was defined as the time elapsed from the date at diagnosis to the occurrence of death. Differences in RFS, EFS and OS between the patients who expressed Ik6 and/or Ik8 isoforms and patients that expressed the other isoforms (Ik1, Ik2, Ik2A, Ik3, Ik3A, Ik4, Ik4A, Ik5 and Ik7) were calculated using the Kaplan-Meier method. A logistic regression analysis was performed to determine the risk of relapse and death in patients with Ik6 and/or Ik8, and different clinical and laboratory characteristics (age, immunophenotype, WBC count, prednisone response). A 95% confidence interval [95% CI] was used, and all tests were considered statistically significant when *p*-value <0.05.

## Results

### Patient characteristics

A total of 113 patients were enrolled for detecting the expression of Ikaros isoforms; 99 patients were diagnosed with B-ALL and 14 with T-ALL. The proportion of female and male patients was roughly equivalent, 41.6% and 58.4% respectively. One patient had Down syndrome. *ETV6-RUNX1* was the gene fusion most frequently observed (12 patients), whereas the poor prognosis *BCR-ABL1* and *MLL-AF9* fusions were detected in five patients (four and one cases, respectively). Six B-ALL patients presented extramedullary disease at diagnosis (four in central nervous system and two in testicles). One patient with T-ALL harbored the *SIL-TAL1* fusion and another case showed mediastinal mass (Table 1). Thirty-nine patients (34.5%) presented adverse events, 29 (25.7%) relapsed, 3 (2.6%) were therapeutic failures, and 7 (6.2%) died (Table 1). Eight (8.1%) patients with B-ALL died by infection (6), thrombosis (1), and pancreatitis (1) (Table 1). Most of the patients (68%) showed at least one of the conventional clinical risk factors, such as age (<1 year or ≥10 years), WBC count (≥50×10^9^/L), presence of the *BCR-ABL1* fusion or *MLL* gene alteration, extramedullary disease, poor prednisone response (PPR) and T cell immunophenotype.

**Table 1:** Characteristics of 113 pediatric patients with ALL.

		Immunophenotype		
		B-ALL n = 99 (%)	T-ALL n = 14 (%)	TOTAL n = 113 (%)
**Gender**	Female	45 (45.5)	2 (14.3)	47 (41.6)
	Male	54 (54.5)	12 (85.7)	66 (58.4)
**Age at diagnosis (years)**	1–9	73 (73.7)	12 (85.7)	85 (75.2)
	<1 or >10	26 (26.3)	2 (14.3)	28 (24.8)
**White blood cell (WBC) count**	< 50x10^9^/L	81 (81.8)	6 (42.8)	87 (77)
	> 50x10^9^/L	18 (18.2)	8 (57.2)	26 (23)
**Fusions**	None	77 (77.8)	13 (92.8)	90 (79.6)
	*ETV6-RUNX1*	12 (12.1)	0	12 (10.6)
	*E2A-PBX1*	5 (5)	0	5 (4.4)
	*BCR-ABL1*	4 (4)	0	4 (3.5)
	*MLL-AF9*	1 (1)	0	1 (0.9)
	*SIL-TAL1*	0	1 (7.2)	1 (0.9)
**Chromosomal abnormalities**	Constitutional trisomy 21	1 (1)	0	1 (0.9)
**Extramedullary disease at diagnosis**	Central nervous system	4 (4)	0	4 (3.5)
	Testicles	2 (2)	0	2 (1.8)
	Mediastinal mass	0	1 (7.1)	1 (0.9)
**Prednisone response**	Good	62 (62.6)	10 (71.4)	72 (63.7)
	Poor	37 (37.4)	4 (28.6)	41 (36.3)
**Adverse events**	None	59 (59.6)	7 (50)	66 (58.4)
	Relapse	23 (23.2)	6 (42.9)	29 (25.7)
	Therapeutic failure	3 (3)	0	3 (2.6)
	Death	6 (6.1)	1 (7.1)	7 (6.2)
	Total adverse events	32 (32.3)	7 (50)	39 (34.5)
**Other deaths by**	Infection	6 (6.1)	0	6 (5.3)
	Thrombosis	1 (1)	0	1 (0.9)
	Pancreatitits	1 (1)	0	1 (0.9)
**Ikaros isoforms**	Other	76 (76.8)	11 (78.6)	87 (77)
	Dominant-negative	23 (23.2)	3 (21.4)	26 (23)
	Ik6	16 (69.6)	0	16 (61.6)
	Ik8	5 (21.8)	3 (100)	8 (30.8)
	Ik6 & Ik8	1 (4.3)	0	1 (3.8)
	Ik8-del*	1 (4.3)	0	1 (3.8)

* Deleted Ik8 variant (Ik8-del). Deletion was confirmed by sequencing analysis.

### Expression of Ikaros isoforms in patients with ALL

Of the 99 patients with B-ALL, 23 expressed Ik6 and/or Ik8 isoforms, 16 of them were Ik6+, 5 were Ik8+, 1 patient expressed both Ik6 and Ik8 isoforms, and 1 patient showed a variant of Ik8 (Table 1). In the last patient, a non-expected band of 300 bp was analyzed by sequencing and the result showed a 30-nucleotide deletion, which represented the loss of 10 amino acids (KSSMPQKFLG) in the exon. According to the alignment to the reference sequences for Ik8 and Ik8 deleted, it was determined that this variant corresponded to Ik8-del. Four patients with Ik6 were positive for gene fusions: *BCR-ABL1* (2/4 patients) *ETV6-RUNX1* (1/12 patients) and *E2A-PBX1* (1/5 patients); the remaining Ik6 positive cases did not have any of the characteristic ALL gene fusions. Only one patient with Ik8 was *ETV6-RUNX1* positive. The remaining B-ALL patients (76) (Table 1) expressed other Ikaros isoforms (Ik1, Ik3A, Ik2, Ik4, Ik5, Ik7, and Ik4A), 17 of them presented gene fusions as follows: *ETV6-RUNX1* (10), *E2A-PBX1* (4), *BCR-ABL1* (2) and *AF9-MLL* (1). Patients with T-ALL only expressed Ik8 (3/14) and none showed gene fusions. Of the 113 patients studied, 23% expressed Ik6 and/or Ik8 and 77% expressed one or more of the other isoforms already reported for Ikaros. Ik1, Ik3A and Ik2 isoforms were predominantly expressed in our patients; Ik4, Ik4A, Ik5 and Ik7 were also expressed although in lower proportion (Table 1).

### Association of Ikaros with adverse events

Of the 26 patients Ik6 and/or Ik8 positive, 9 (34.6%) presented adverse events, 14 (53.8%) did not present events and 3 (11.5%) died by infection.

Of the 9 patients with adverse events 3 expressed exclusively Ik6 and 6 patients expressed Ik6, Ik8 and other isoforms. Six out of the 9 patients were Ik6 positive (4 relapsed, 1 presented therapeutic failure and 1 died), 3/9 were Ik8 positive (2 showed relapsed and 1 died) [S1 Fig]. Of the 14 patients who did not present adverse events, 6 expressed exclusively Ik6 and 1 Ik8, the 7 remaining patients also expressed the other Ikaros isoforms as follows: Ik1 and Ik5/Ik7 (in 1 patient each), Ik3A and Ik2 (3 patients), Ik4 (4 patients) and Ik4A (5 patients) (S1 Fig). The group with DNI also expressed long and short isoforms, showing 22 different expression patterns. A similar behavior was observed between patients without adverse events and patients with relapse. Patients who died expressed all Ikaros isoforms; they did not express exclusively DNI (S1 Fig).

Of the 87 patients who expressed other Ikaros isoforms, 26 (29.9%) presented adverse events (23 relapsed, 2 had therapeutic failures and 1 died); the long isoforms Ik1, Ik2 and Ik3A were predominantly expressed in patients with relapse.

The univariate analysis revealed that the DNI expression was not statistically associated with relapse (*p* = 0.803), death (*p* = 0.452) or prednisone response (*p* = 1.0) (Table 2); in contrast, the analysis of conventional risk factors revealed that the age and the WBC count were significantly associated with death, and the WBC count also influenced the relapse and prednisone response (Fisher's exact test *p*<0.05, Table 2).

**Table 2:** Clinical-biological characteristics and Ikaros isoforms expression in ALL children and their association with relapse, death or prednisone response.

		Relapse			Death			Prednisone response		
Features	Total number (%)	Yes	Non	*p*	Yes	Non	*p*	Poor	Good	*p*
**Age (years)**										
1 < 9	85 (75.2)	22	63	1.000	17	68	0.003*	32	53	0.656
1 < or > 10	28 (24.8)	7	21		14	14		9	19	
**Gender**										
Male	66 (58.4)	17	49	1.000	17	49	0.672	26	40	0.435
Female	47 (41.6)	12	35		14	33		15	32	
**WBC count**										
< 50 x 10^9^/L	87 (77)	18	69	0.039*	18	69	0.005*	27	60	0.039*
> 50 x 10^9^/L	26 (23)	11	15		13	13		14	12	
**Immunophenotype**										
B-ALL	99 (87.6)	23	76	0.186	26	73	0.525	37	62	0.767
T-ALL	14 (12.4)	6	8		5	9		4	10	
**Ikaros isoforms**										
Others	87 (77)	23	64	0.803	22	65	0.452	32	55	1.000
Ik6 and/or Ik8	26 (23)	6	20		9	17		9	17	

*p* = Analyzed by Fisher exact test.

* = Statistically significant *p* value.

The expression of Ik6 and/or Ik8 was not statistically associated with lower EFS (*p* = 0.667), and RFS (*p* = 0.432); since EFS was identical in patients who expressed DNI and patients with other isoforms (in both cases, 50%). A similar behavior was observed in RFS, the survival was 59% in patients with Ik6 and/or Ik8 and 57% in patients with other isoforms (S2A and S2B Fig). OS analysis demonstrated that patients who expressed Ik6 and/or Ik8 tended to have lower survival (58%) compared to those who expressed other isoforms (66%), although this result was not statistically significant (*p* = 0.531) (S2C Fig).

In our patients, the expression of Ik6 and Ik8 did not increase the risk of relapse (*p* = 0.731) or death (*p* = 0.352) (univariate logistic regression *p*<0.05, Table 3). The risk of relapse was significantly increased by the high WBC count (*p* = 0.030; OR 2.811, 95% CI 1.103–7.161) and PPR (*p* = 0.016; OR 3.905, 95% CI 1.219–6.920); the risk of death was influenced by the age <1 year or ≥10 years (*p* = 0.003; OR 4.0, 95% CI 1.607–9.954) and the high WBC count (*p* = 0.005; OR 3.833, 95% CI 1.516–9.690) (Table 3). In these patients the T cell immunophenotype did not mean a risk factor of relapse or death, although belonging to high-risk group.

**Table 3:** Risk analysis for clinical-biological characteristics and dominant-negative Ikaros isoforms expression.

	Relapse OR (IC 95%)	*p*	Death OR (IC 95%)	*p*
**Age (years)**				
<1 or > 10	0.095 (0.357–2.552)	0.926	4.000 (1.607–9.954)	0.003*
**Immunophenotype**				
T-ALL	2.478 (0.779–7.880)	0.124	1.560 (0.479–5.083)	0.461
**WBC count**				
> 50x10^9^/L	2.811 (1.103–7.161)	0.030*	3.833 (1.516–9.690)	0.005*
**Prednisone response**				
Poor	3.905 (1.219–6.920)	0.016*	1.678 (0.721–3.902)	0.230
**Dominant-negative isoforms**				
Ik6 and/or Ik8	0.835 (0.298–2.337)	0.731	1.564 (0.610–4.010)	0.352

*p* = Analyzed by Fisher exact test.

* = Statistically significant *p* value.

It was not possible to define an expression pattern of Ikaros isoforms associated with the presence or absence of adverse events (S1 Fig). Sixteen of these 26 patients presented one or more high risk factors including age, high WBC count, PPR, fusions *MLL-AF9* or *BCR-ABL1*, extramedullary disease and T cell immunophenotype. In the standard and high risk groups the presence of Ik6 and/or Ik8 did not significantly decreased the EFS and RFS (S3A, S3B, S3C and S3D Fig). However, the patients who harbored these isoforms tended to have decreased OS in both standard (69%) and high (46%) risk groups compared to patients without DNI (82.5% and 59% respectively), although this association was not statistically significant (*p* = 0.503 and *p* = 0.387 respectively) (S3E and S3F Fig).

**Fig 1:**
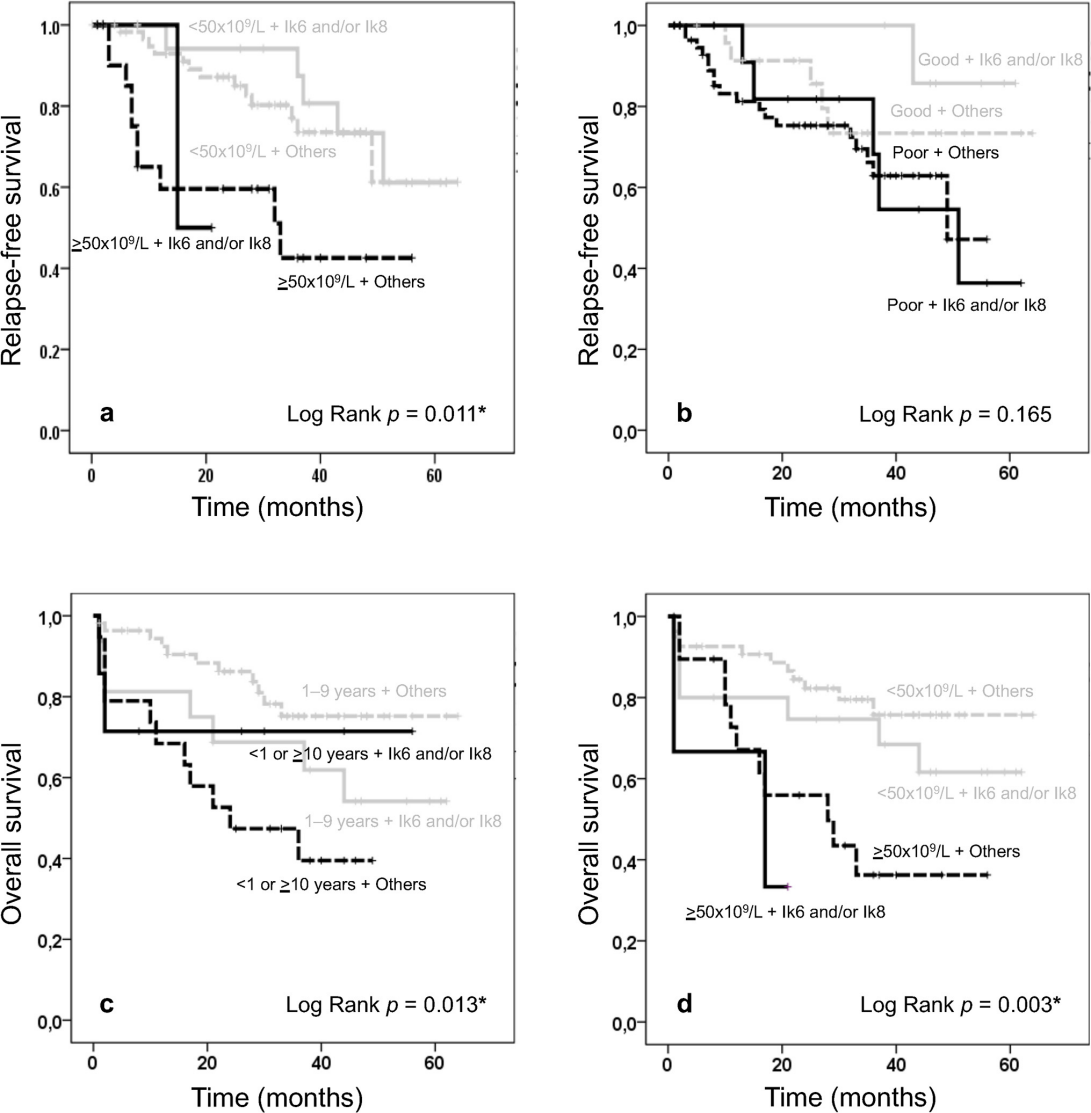
Kaplan Meier curves according to clinical risk. The clinical risk features as WBC count (a & d), prednisone response (b) and age (c) were evaluated according to the expression of Ik6 and/or Ik8 (solid gray and black line) or other Ikaros isoforms (dotted gray and black lines). We observed statistical difference for age and WBC count.

A survival analysis was performed for the expression of Ik6 and/or Ik8 isoforms in combination with the clinical high-risk features that showed statistically significance by the univariate logistic regression analysis (Table 3). The WBC count revealed association with lower RFS (*p* = 0.011; Fig 1A) and the prednisone response did not (*p* = 0.165; Fig 1B). The age and the WBC count showed association with lower OS (*p* = 0.013 and *p* = 0.003; Fig 1C and 1D respectively). The DNI expression influenced the OS in the group of patients classified as standard risk by age (1–9 years old) and WBC count (<50x10^9^/L), since the survival was lower in patients that expressed Ik6 and/or Ik8 compared with those that expressed other Ikaros isoforms (Fig 1C and 1D).

## Discussion

### Frequency of Ik6 and Ik8 isoforms in the ALL patients

The frequency of the DNI (23%; Ik6 with 15% and Ik8 with 8%) found in our population was within the range (10% to 27%) of values reported for children with ALL [5, 10, 12–14, 18, 19]. The frequency variations could be related to the methodologies used to identify mutations and/or deletions in *IKZF1* [8]. Although there is a correlation between the presence of different isoforms and genomic deletions in *IKZF1* [12], in this study the mutations were not detected. In addition, it is possible that yet undescribed, non-functional isoforms were left out of the analysis. In a similar study and using the same methodology, the Ik6 was present in 15% of ALL-children [13]. Same authors reported that the incidence of *BCR-ABL1* was higher (14%) than that found in Western cohorts (3–5%) [11,13], in consequence 43% of their patients were Ik6 positive; furthermore, they also showed a statistical correlation between the existence of both Ik6 and *BCR-ABL1* fusion [13]. We only detected 3.5% of our patients with the *BCR-ABL1* fusion, however half of them were Ik6 positive. In patients with B-ALL, the Ik8 isoform was less frequent than Ik6, and in cases with T-ALL, Ik6 was not observed. Meleshko and colleagues also reported that the frequency of Ik6 is lesser in T-ALL (6%) than in B-ALL (12%) [6]. To confirm whether the frequency of Ik6 is lower or definitely absent in our T-ALL patients, an increase in sample size would be required. Only one variant isoform was identified in this study, it was present in a B-ALL patient and it corresponded to the Ik8-del.

The long isoforms (Ik1, Ik3A and Ik2) were expressed more frequently in our patients in contrast to other populations reported that express both short and long isoforms in B-ALL cases and only short isoforms in the T-ALL patients [6]. Much like other authors [6,20–22], we observed a great heterogeneity in the expression of all Ikaros transcripts in the ALL-children studied.

### Ik6 and Ik8 expression and their association with EFS, RFS and OS

In this study, 61.5% of the patients positive for Ik6 and/or Ik8 showed one or more conventional risk factors. Patients with DNI have been associated with increased frequency of relapse compared to those with other isoforms [13]; in our study, the frequencies of adverse events and relapses were similar in patients who expressed DNI (34.6% and 23.1% respectively) compared to patients with other Ikaros isoforms (34.5% and 26.4% respectively). After the follow-up period of 60 months, relapses were more frequent in our patients than those reported by other groups (28% vs. 15%, respectively) [1]. No statistically significant differences in the RFS, EFS, OS were observed between patients with other isoforms and in patients with Ik6 and/or Ik8.

Univariate analysis revealed that the occurrence of relapse and death events were affected by high WBC count, initial PPR and age at risk, but not by the presence of DNI. However, the RFS and OS decreased in patients with Ik6+ and/or Ik8+, PPR, and high WBC count. Ik6 and Ik8 could be considered as risk markers of adverse events in the initial stratification, only if their assessment is combined with conventional risk factors. Recently some authors have reported that *IKZF1* deletions were not significantly associated with RFS and EFS [14,23]. Similar to these studies, in our patients, the DNI were only associated with OS, although it is important to consider that infections, thrombosis and pancreatitis were present in 60% of death patients.

The role of *IKZF1* alterations as a prognostic marker of relapse and death in ALL-children has been controversial [14,24]. It is important to note that our patients were treated with the protocol established by the PMIP, which is based on the St Jude XIIIb, and is applied widely in Mexican ALL patients who do not have access to other medical social security systems. It has been suggested that patients with *IKZF1* alterations could benefit from intensive therapy with L-asparaginase to decrease relapses and thus improve outcome [14]. This proposal could be considered for our population based on the high number of patients positive for the DNI. However, it is important to note that in addition to deaths caused by relapse, induction deaths also occurred, which could be related to other causes besides the response to treatment. Because minimal residual disease analysis is not accessible to all patients enrolled in the PMIP, it is essential for us to identify cases prone to relapse through potential risk markers such as the DNI of *IKZF1* [17]. However, we did not find *IKZF1* as an independent marker of relapse. It is important to consider that gene mutations were not analyzed in this study and they are relevant in the *IKZF1* assessment as a prognostic marker [25].

Even without analyzing mutations, we identified the DNI in 23% of patients, and most of these patients (65.4%) did not experience relapse and death. As has been suggested, the detection of alterations in the genes of JAK-STAT5 pathway, *JAK2* and *CRLF2*, together with the analysis of *IKZF1*, would likely increase the possibility of obtaining a genetic signature to identify cases prone to relapse [24]. Another possibility is to consider that other alterations as *ERG* gene deletions (*ERG* ^del^) confer a favorable outcome. Recently it has been demonstrated that patients with deletions in both *IKZF1* and *ERG* genes, showed a better EFS (85.7%) compared to those who only had *IKZF1* deletions (51.3%) [23,26].

Considering that several patients showed mixed expression profiles of Ikaros isoforms, and some of them did not have relapse despite expressed DNI, it is possible that the Ikaros function in patients Ik6+ and/or Ik8+ might be compensated by the expression of other Ikaros isoforms [22]. The functional and biological significance of the DNI (Ik6 and Ik8) was not evaluated in this study. However, as an attempt to evaluate indirectly the function of these isoforms we analyzed the co-expression of myeloid markers (CD13, CD15 and MPO) from the patient’s immunophenotype at diagnosis. This analysis was based on the dual role of Ikaros in hematopoiesis; it promotes the differentiation of the lymphoid lineage, but also suppresses the myeloid development [27,28]. In particular, Ikaros represses the expression of the *Fucosyltransferase-4* gene, preventing the synthesis of CD15 [6]. If Ikaros is absent or non-functional, CD15 could be co-expressed in the lymphoid leukemic blasts. The immunophenotype showed that none of the patients with DNI co-expressed myeloid markers (data not shown). In this context, we suggest that the function of Ikaros was not affected by the expression of Ik6 and Ik8. This result is in accordance with data reported by other authors, who demonstrates that the function and expression of Ikaros were not decrease by *IKZF1* deletions in ALL blasts [22].

The expression patterns of isoforms in patients with DNI, did not show relevant differences between patients without adverse events and patients with relapse. These findings suggest that the expression of Ik6 and Ik8 is not a prognostic marker of relapse at diagnosis; therefore they could not be used in the initial risk stratification. In this context, it is important to consider other mechanisms that could interfere with the presence of adverse events such as the accumulation of alterations in different genes and the clonal heterogeneity in the leukemic cells. All these characteristics could influence the presence and number of relapses or death [9,22,29].

In conclusion, this study is one of the first to evaluate stratification markers in patients treated with a single Mexican PMIP. Based on the results obtained in our patients, Ik6 and Ik8 do not represent an independent risk marker for relapse or death; therefore in our population they are not useful in the initial risk stratification.

## Supporting Information

S1 Fig Heterogeneity in the expression of Ikaros isoforms in patients with Ik6 and/or Ik8. A great diversity of Ikaros isoforms patterns (22) were observed in ALL-children. Patients with DNI were classified according to presence or absence of adverse events (without events, with relapses and deaths), and according to expression levels (high, medium, low or null). DNI: Dominat-negative isoforms; ZFD: Zinc finger domains; *Deaths by infection.
(TIF)

S2 Fig Survival analysis according to the Ikaros isoforms expression. The Kaplan Meier curves of EFS (a), RFS (b) and OS (c) show survival of the ALL children with Ik6 and/or Ik8 (solid black line) or others isoforms (dotted black line). No statistically significant difference was found.
(TIF)

S3 Fig Survival analysis according to risk group. The Kaplan Meier curves for EFS (a & b), RFS (c & d) and OS (e & f) show that the patients classified into standard (a, c & e) and high (b, d & f) risk groups expressed Ik6 and/or Ik8 (solid black line) and others Ikaros isoforms (dotted black line). No statistically significant difference was found, but the expression of the dominant-negative isoforms tended to decrease OS in both groups.
(TIF)
