# Supplementary figures and images for: Expression of Ik6 and Ik8 Isoforms and Their Association with Relapse and Death in Mexican Children with Acute Lymphoblastic Leukemia
Source: PLoS One. 2015 Jul 1;10(7):e0130756. doi: 10.1371/journal.pone.0130756 (PMC4488851; doi:10.1371/journal.pone.0130756)

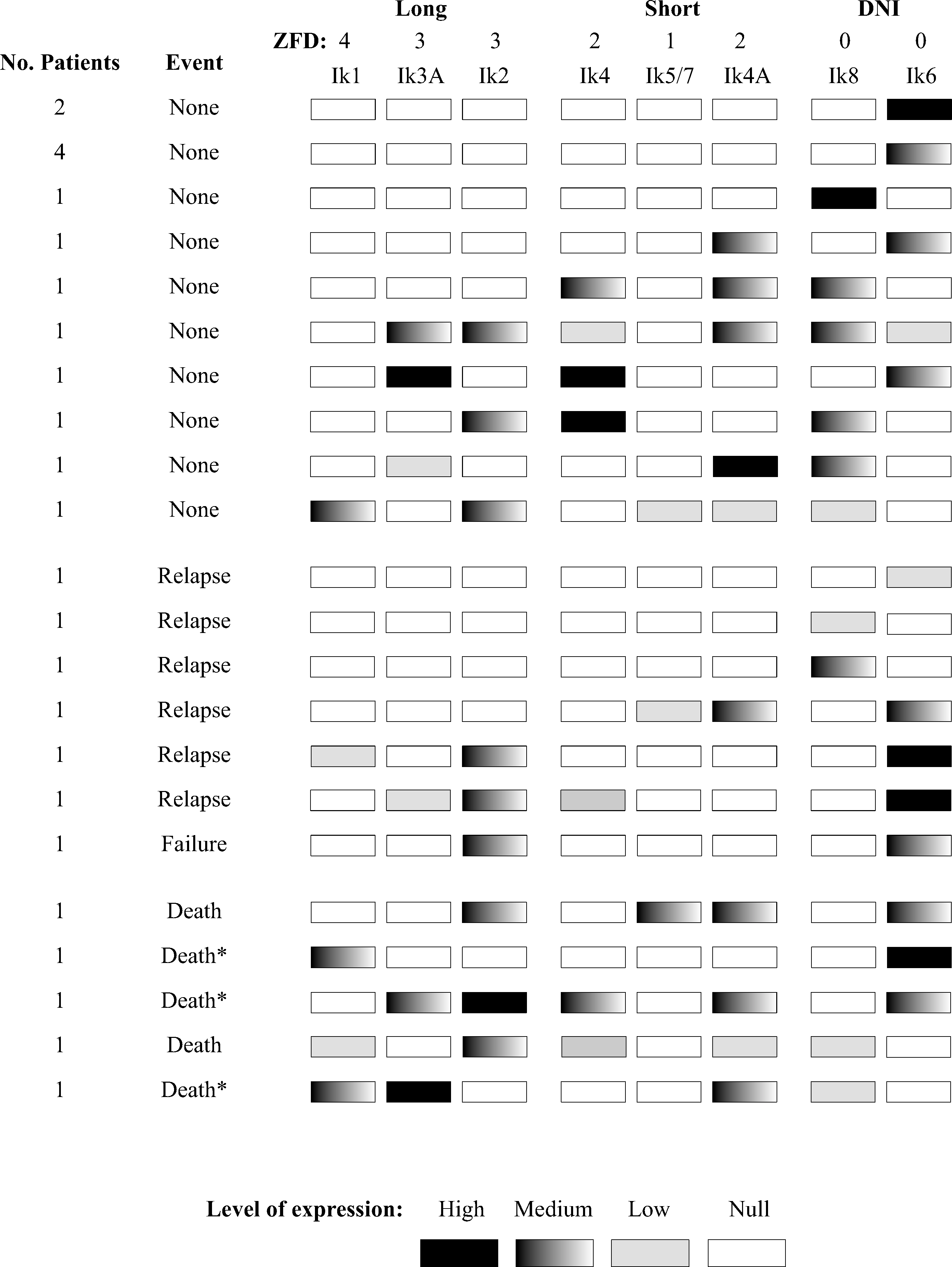

Supplement: S1 Fig — A great diversity of Ikaros isoforms patterns (22) were observed in ALL-children. Patients with DNI were classified according to presence or absence of adverse events (without events, with relapses and deaths), and according to expression levels (high, medium, low or null). DNI: Dominat-negative isoforms; ZFD: Zinc finger domains; *Deaths by infection. (TIF) [file pone.0130756.s001.tif]

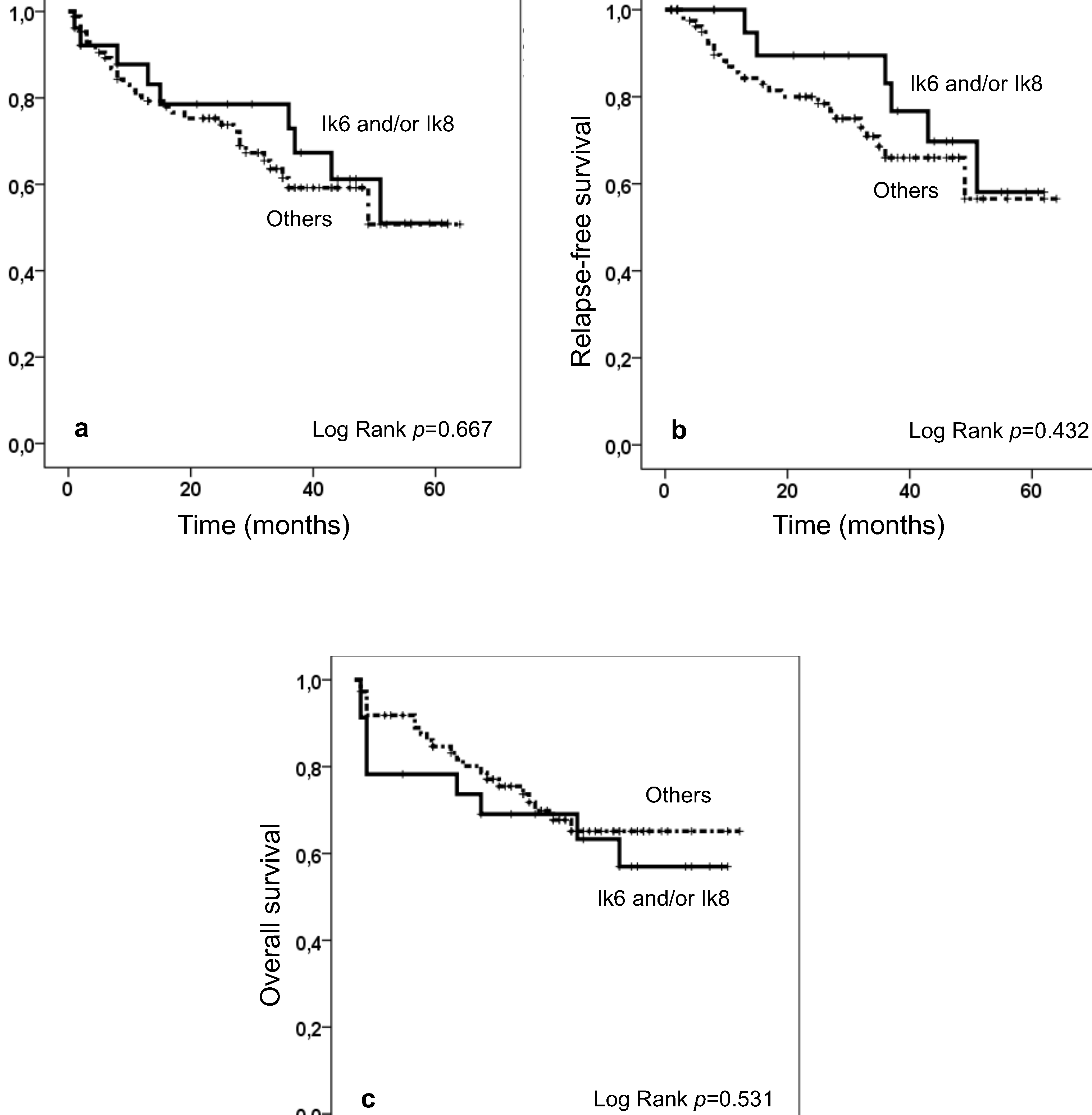

Supplement: S2 Fig — The Kaplan Meier curves of EFS (a), RFS (b) and OS (c) show survival of the ALL children with Ik6 and/or Ik8 (solid black line) or others isoforms (dotted black line). No statistically significant difference was found. (TIF) [file pone.0130756.s002.tif]

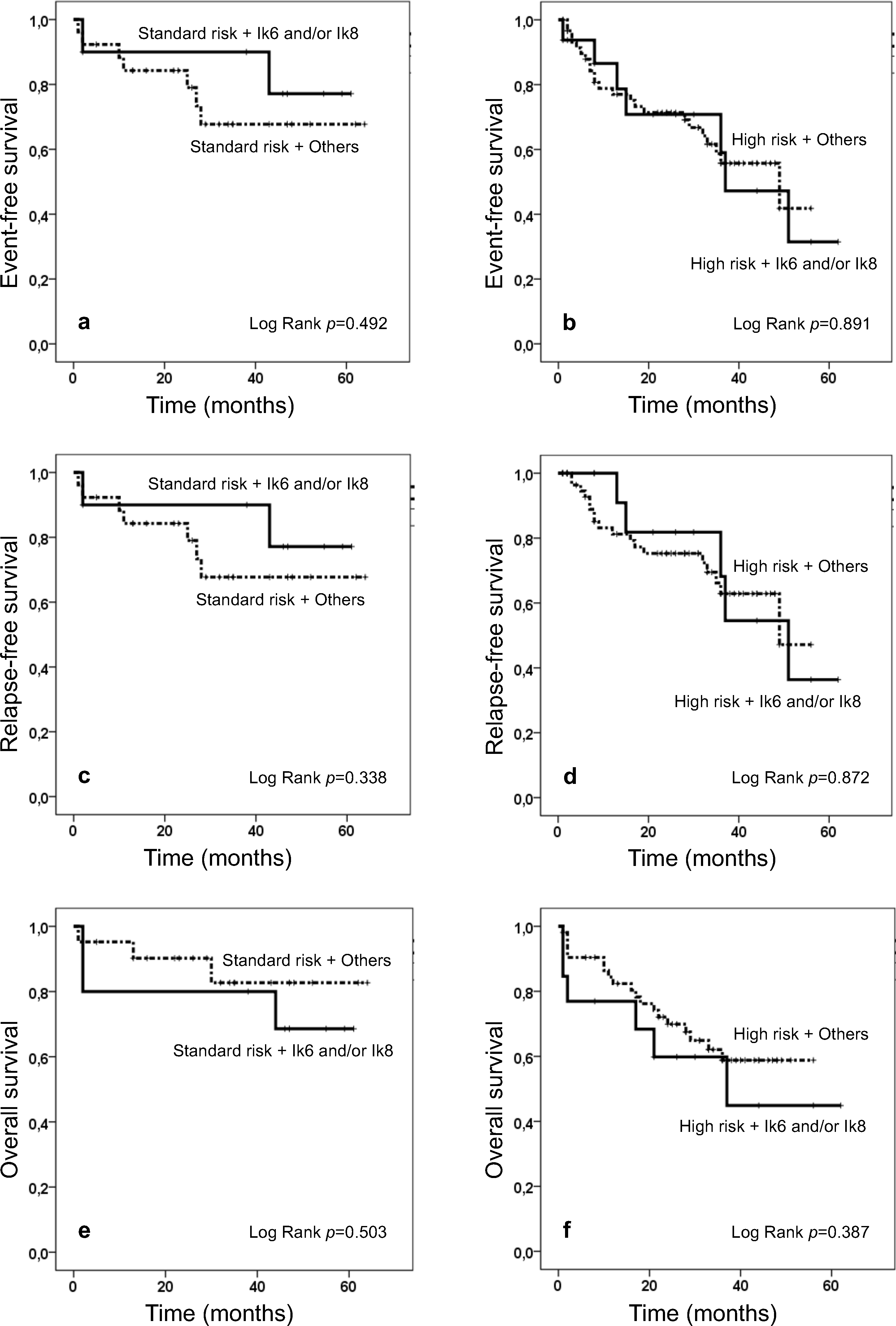

Supplement: S3 Fig — The Kaplan Meier curves for EFS (a & b), RFS (c & d) and OS (e & f) show that the patients classified into standard (a, c & e) and high (b, d & f) risk groups expressed Ik6 and/or Ik8 (solid black line) and others Ikaros isoforms (dotted black line). No statistically significant difference was found, but the expression of the dominant-negative isoforms tended to decrease OS in both groups. (TIF) [file pone.0130756.s003.tif]
